# *Mycoplasma gallisepticum* Infection Impaired the Structural Integrity and Immune Function of Bursa of Fabricius in Chicken: Implication of Oxidative Stress and Apoptosis

**DOI:** 10.3389/fvets.2020.00225

**Published:** 2020-04-24

**Authors:** Wei Zhang, Yuhao Liu, Qiaomei Zhang, Syed Waqas Ali Shah, Zhiyong Wu, Jian Wang, Muhammad Ishfaq, Jichang Li

**Affiliations:** ^1^Heilongjiang Key Laboratory for Animal Disease Control and Pharmaceutical Development, College of Veterinary Medicine, Northeast Agricultural University, Harbin, China; ^2^Department of Animal Nutrition, College of Animal Science and Technology, Northeast Agricultural University, Harbin, China

**Keywords:** immune damage, chickens, bursa of fabricius, apoptosis, oxidative stress, *Mycoplasma gallisepticum*

## Abstract

*Mycoplasma gallisepticum* (MG) induces a dysregulated immune response in the lungs and air ways of poultry. However, the mechanism of MG-induced immune dysregulation is still not completely understood. In the present study, the effect of MG-infection on chicken bursa of fabricius (BOF) is investigated. Histopathology, electron microscopy, TUNEL assay, qRT-PCR and western blot were employed to examine the hallmarks of oxidative stress and apoptosis. The data revealed that MG-infection induced oxidative stress and decreased antioxidant responses in BOF tissues compared to control group. Histopathological study showed pathological changes including reduction in lymphocytes and increased inflammatory cell infiltration in MG-infection group. Ultrastructural assessment represents obvious signs of apoptosis such as mitochondrial swelling, shrinkage of nuclear membrane and fragmentation of nucleus. Increased cytokine activities were observed in MG-infection group compared to control group. Meanwhile, the mRNA and protein expression level of apoptosis-related genes were significantly (*p* < 0.05) upregulated in MG-infection group. Terminal deoxynucleotidyl transferase-mediated dUTP nick end-labeling (TUNEL) assay further confirmed that MG induced apoptosis in BOF tissues as TUNEL-stained positive nuclei were remarkably increased in MG-infection group. In addition, MG-infection significantly reduced the number of CD8^+^ lymphocytes in chicken BOF at day 7. Moreover, bacterial load significantly increased at day 3 and day 7 in MG-infection group compared to control group. These results suggested that MG-infection impaired the structural integrity, induced oxidative stress and apoptosis in chicken BOF tissues, which could be the possible causes of damage to immune function in chicken BOF.

## Introduction

*Mycoplasma gallisepticum* (MG) belongs to the class *Mollicutes* and the family *Mycoplasmataceae* (1). MG is an important avian pathogen that mainly infect chickens' respiratory tract and causes chronic inflammation in airway passages and lungs. Previous studies demonstrated that MG is the primary cause of chronic respiratory disease in chickens and infectious sinusitis in turkeys, characterized by nasal discharge, sneezing, and coughing (2, 3). Researchers reported that MG-infection causes significant economic losses to the poultry industry including the reduction in egg production, hatchability, and low meat quality (4–6). MG-infection induces respiratory distress, profound inflammatory response in mucosal tissues and immune dysregulation in chickens (4). Moreover, the property of cytoadherence of MG produced additional virulence factors including superoxide radicals leading to immune dysregulation (4, 7). However, the mechanism underlying immune dysregulation induced by MG-infection is still unclear.

Studies demonstrated that pathogen infections cause imbalance in cells normal homeostasis, damage mitochondria, release of mitochondrial DNA, and produced reactive oxygen species (ROS) (8). In addition, both chronic and acute inflammation results in significant alterations in redox equilibrium *in vivo* which is associated with increased oxidant generation during infections (9, 10). Cells develop special mechanisms such as the initiation of inflammatory responses to overcome oxidative stress, inhibit tissue damage, and disease development (11). But, excessive ROS accumulation results in tissue impairment, DNA fragmentation, damage lipids, and proteins, and could induce apoptosis (12, 13). Accumulating evidences showed that apoptosis is the representative phenomenon by which ROS causes cell death (14). Researchers demonstrated that excessive ROS accumulation involves the triggering of immune organs to undergo deviant apoptosis associated with the abnormal function of immune organs (15). However, the effect of MG-infection on chicken immune function, especially the molecular mechanism is not studied in detail.

The bursa of fabricius (BOF) is a humoral immune organ that plays a critical role in innate immune responses and is a place for B-lymphocyte maturation and differentiation (16). B-lymphocytes then migrate to peripheral lymphoid organs to colonize and orchestrate functional immune responses that fight against infections (17), and release proinflammatory cytokines including tumor necrosis factor alpha (TNF-α), interleukein-1 β (IL-1β), IL-6, IL-8, and IL-4 to develop effective inflammatory responses (18). It has been reported that the increase level of these cytokines could affect Th1/Th2 ratio and impair the function of antigen presenting cells leading to immune disorder (19). Researchers reported that MG-mediated inflammatory responses in airway passages and lungs of chickens, but it is still unclear whether MG-infection alters cytokines expression in chicken BOF and affect its normal functioning.

The main objective of the present study was to investigate the effect of MG-infection on chicken BOF. The research will provide a better understanding of the dysregulated immune responses caused by MG-infection and laid a foundation for further comparative medicinal studies to prevent MG-induced immune impairment in chicken BOF.

## Materials and Methods

### Ethical Statement

The present study was conducted under the approval of Laboratory Animal Ethics Committee of Northeast Agricultural University (Heilongjiang province, China) in accordance with Laboratory Animal-Guideline for ethical review of animal welfare (GB/T 35892-2018, National Standards of the People's Republic of China).

### MG and Culture Conditions

MG strain R_low_ were provided by Harbin Veterinary Research Institute (Chinese Academy of Agricultural Science, Heilongjiang, China). Modified Hayflicks medium was used to culture MG as mentioned previously (1). A color change was observed from red to orange when MG reached to its mid-exponential phase. MG at a density of 1 × 10^9^ CCU/ml (color change unit per milliliter) were employed in the study to challenge chickens.

### Experimental Chickens, Infection and Sample Collection

White Leghorn chickens were purchased from Chia Chau chicken farm (Harbin, Heilongjiang, China). The chickens were randomly divided into two groups (10 chickens/group) in three replicates; control group and MG-infection group. Chickens were in healthy conditions, MG and *Mycoplasma synoviae*-free and did not undergo vaccination. Prior infection, chickens were screened for MG and *M. synoviae* using serum plate agglutination (China Veterinary Drug Administration, Beijing, China). Chickens were reared for 1 week prior of experiments to adapt to experimental conditions and provided fresh drinking water and adlibitum feed, and infected with MG strain R_low_ at a concentration of 1 × 10^9^ CCU/mL in the bilateral air sacs as described previously (20). Chickens from each experimental group were sacrificed after 1-, 3- and 7-days post-infection. The whole bursa was collected and a fresh piece was processed for antioxidant and cytokine activities, histopathological and ultrastructural examination, and the remaining samples were stored at −80°C for further experimental analyses.

### Determination of Oxidative Stress-related Parameters

Malonaldehyde (MDA, Cat no. A003-1), gamma glutamyl transferase (γ-GT, Cat no. C017), glutathione (GSH, Cat. No. A006), catalase (CAT, Cat no. A007-1), superoxide dismutase (SOD, Cat no. A001-1), and glutathione peroxidase activities (GSH-Px, Cat no. A006) were determined in fresh bursal samples according to manufacturer's instructions. All the kits were purchased from Nanjing Jiancheng Bioengineering Institute (Nanjing, China). Samples were weighed, dissolved in nine-fold volume of physiological saline solution, and homogenized at 4°C. The homogenized samples were then centrifuged at 1000 × g for 10 min, and the supernatant was collected and examined for the above-mentioned oxidative stress-related parameters on a microplate spectrophotometer (Philes, Nanjing, China).

### Histopathological and Ultrastructural Observation

A piece of bursal sample was processed for histopathological examination as described previously (21). In brief, samples were fixed in 10% formalin for 12 h, dehydrated in a series of ethanol solution, embedded in wax and cut into tiny sections for mounting on glass slides. Hematoxylin and eosin staining were carried out and the slides were then examined under a light microscope (Nikon E100, Japan). For ultrastructural examination, specimens were first fixed in glutaraldehyde (2.5%) and rinsed twice in phosphate buffer (0.2 M, pH = 7.2) for 15 min. After the samples were incubated in osmium tetroxide (1%), dehydrated in alcohol and embedded in epoxy resin, and stained with uranyl acetate. The stained tiny sections were then observed under transmission electron microscope (JEOL Ltd., Tokyo, Japan, GEM-1200ES).

### Measurement of Cytokine Activities

Cytokines activities including TNF-α (kit no. E-80035), IL-6 (kit no. E-80100), IL-8 (kit no. E-75013) and IL-1β (kit no. E-75004) were determined by ELISA assays according to manufacturer's instructions (He Guo Biological Co., Ltd. Shanghai, China). Samples were homogenized, centrifuged (1000 × g for 10 min), and supernatant was collected. Standards (50 μl) and samples (10 μl) were added to 96 well-coated microplate. Then, 40 μl diluent was added to those wells containing sample solution, followed by the addition of horseradish peroxidases (HRP)-conjugate and incubated at 37°C for 1 h. The solution was decanted from each well and the plate was washed for 5 times. After adding chromogenic substrate (100 μl), the plate was incubated at room temperature in dark for 15 min. Next, stop solution (100 μl) was added to terminate the reaction. Besides blank control, all the samples were run in duplicate and readings were taken on an iMARKTM microplate reader (Bio-Rad Co., Ltd. Shanghai, China) at a wavelength of 450 nm.

### Detection of NO Content and iNOS Activity

The content of nitric oxide (NO, Cat. No. A014-1) and inducible nitric oxide synthase (iNOS, Cat. No. A013) activity were measured by ELISA assays according to the instructions of the kit. The kits were obtained from Nanjing Jiancheng Bioengineering Institute (Nanjing, China). Supernatant was collected from experimental samples as discussed in the above section. Readings were taken in duplicate along with a blank control, and NO content and iNOS activity were determined in the two experimental groups.

### Quantitative Real-time Polymerase Chain Reaction (qRT-PCR)

TRIzol reagent was employed to extract total RNA from BOF tissues according to manufacturer's instructions (Thermo Scientific, Shanghai, China) and the concentration of RNA was determined at 260/280 ratio by using a NanoDrop spectrophotometer (2000c, Thermo Scientific, Shanghai, China) as stated previously (22). RNA samples of optical density (OD) values ranging from 1.81 to 1.96 were selected for first strand cDNA synthesis. The PrimeScript™ RT reagent Kit with gDNA Eraser for cDNA synthesis was purchased from Takara Company Ltd., (Code No. RR047A, Dalian, China). The gDNA eraser (DNase) was used to remove genomic DNA from the samples. qRT-PCR was carried out in a Roche LightCycler instrument (Shanghai, China) by using a kit obtained from Takara Company Ltd., (catalog no. RR820A, Dalian, China). The primers of the target genes and β-actin are shown in Table 1. The mRNA of all genes was analyzed and quantified by the method as explained earlier (23).

**Table 1:** List of primers used in RT-PCR.

**Target gene**	**Primers (from 5^**′**^ to 3^**′**^)**	**Length**
iNOS2	Forward 5′- GAAGTGGTATGCTCTGCCTGCTG-3′ Reverse 5′- GTCTCGCACTCCAATCTCTGTTCC-3′	115
TNF-α	Forward 5′- TGATCGTGACACGTCTCTGC-3′ Reverse 5′- CAACCAGCTATGCACCCCAG-3′	88
IL-6	Forward 5′- TTCACCGTGTGCGAGAACAGC-3′ Reverse 5′- CAGCCGTCCTCCTCCGTCAC-3′	80
IL-1β	Forward 5′- AGCAGCCTCAGCGAAGAGACC-3′ Reverse 5′- GTCCACTGTGGTGTGCTCAGAATC-3′	90
Bax	Forward 5′- ACTCTGCTGCTGCTCTCCTCTC-3′ Reverse 5′- ATCCACGCAGTGCCAGATGTAATC-3′	174
Caspase-3	Forward 5′- TACCGGACTGTCATCTCGTTCAGG-3′ Reverse 5′- ACTGCTTCGCTTGCTGTGATCTTC-3′	166
Caspase-8	Forward 5′- GGAAGCAGTGCCAGAACTCAGAAG-3′ Reverse 5′- TTGTTGTGGTCCATGCACCGATAG-3′	174
Caspase-9	Forward 5′- CCGAAGGAGCAAGCACGACAG-3′ Reverse 5′- CATCTAGCATGTCAGCCAGGTCAC-3′	121
P53	Forward 5′- GGAGATGGAACCATTGCTGGAACC-3′ Reverse 5′- GCTCCTGCCAGTTGCTGTGATC-3′	113
Bcl2	Forward 5′- GAGTTCGGCGGCGTGATGTG-3′ Reverse 5′- TTCAGGTACTCGGTCATCCAGGTG-3′	92
Cytochrome C	Forward 5′- CCTAATCGCCGTGGCCTTCTTAAC-3′ Reverse 5′- GGAGGAGGTAGATGGTCGGATTGG-3′	163
β-actin	Forward 5′- CAACACAGTGCTGTCTGGTGGTAC-3′ Reverse 5′- CTCCTGCTTGCTGATCCACATCTG-3′	199

### Immunoblotting

Protein extraction was performed by using radioimmunoprecipitation assay (RIPA) and a protease inhibitor phenylmethyl sulfonyl fluoride (PMSF) from chicken BOF tissues (24), with a few modifications. In brief, samples were weighed and the required amount of RIPA plus PMSF was added to samples in eppendorf tubes. The samples were then lysed by a tissue lyser machine (Shanghai, China). The total proteins were collected in a new centrifuge tubes and a quarter of 5 × SDS loading buffer was added, mixed and boiled for 5 min. Equal amount of proteins were separated on separating gel (10–15%) and transferred to nitrocellulose membrane. Membranes were blocked at room temperature with 5% skimmed milk TBST solution for 1 h. Primary antibodies including anti-β-actin (1:1000), anti-iNOS (1:1000), anti-Caspase-3 (1:2000), anti-Caspase-9 (1:3000), anti-Bax (1:500), anti-Cytochrome-C (1:800) and anti-Bcl-2 (1:500) were diluted and dissolved in TBST and incubated overnight with nitrocellulose membranes. After, the membranes were washed thrice and again incubated with secondary anti-mouse (1:3000) or anti-rabbit (1:3000) IgG horseradish peroxidases for 1 h at room temperature. β-actin and secondary anti-mouse or anti-rabbit antibodies were bought from Bioss Antibodies Inc. (Beijing, China). All other antibodies were bought from Wanleibio Co., Ltd. (Shanghai, China). The bands were visualized with enhance chemiluminescence (ECL) reagent (Beyotime, China).

### Terminal Deoxynucleotidyl Transferase–mediated dUTP Nick Endlabeling (TUNEL) Assay

To assess apoptosis in BOF tissues during MG infection, TUNEL assay [Beyotime biotechnology Ltd., (Jiangsu, China)] was employed to determine the level of apoptotic cells as described earlier (25). TUNEL assay is a standard technique for the detection of DNA fragmentation, and the assay was carried out according to the manufacturer's instructions under a fluorescence microscope. In brief, BOF tissues were first fixed in 10% formalin, dehydrated in graded ethanol series and embedded in paraffin wax. The slides were cut into tiny sections and mounted on glass slides. After, 3% hydrogen peroxide was used to inhibit the endogenous peroxidase activity, rinsed in distill water, counterstained with hematoxylin, and dehydrated with a series of alcohol solution. Finally, positive stained apoptotic nuclei were observed under the microscope.

### Immunofluorescence Microscopic Assay

In order to determine the effect of MG-infection on chicken immune responses, the number of CD8^+^ lymphocytes were determined by immunofluorescence microscopy as described earlier (26). In brief, bursal samples were first incubated at 4°C with anti-CD8^+^ antibody (1:500) for 12 h in blocking solution following incubation with goat anti-serum at room temperature. Then, the samples were incubated with CY3 labeled anti-rabbit IgG (1:300, Sinobiological Co. Ltd., Beijing, China). After, the sections were mounted with DAPI (Beyotime Biotechnology, Co, Ltd., Jiangsu, China) and examined under an inverted microscope (Nikon TE2000).

### Quantification of MG Load in BOF by qRT-PCR

DNA copies of MG was identified in chicken BOF by using qRT-PCR with a Roche LightCycler instrument (Roche, Shanghai, China) as described previously (27). MG specific primers MG14F (50-GAG CTA ATC TGT AAA GTT GGTC-3′; melting temperature [Tm] 57.80°C) and MG13R (50-GCT TCC TTG CGG TTA GCA AC-3′; Tm 63.6°C) were used in the method as stated earlier (28). DNA template was extracted with a bacterial DNA kit purchased from Omega Bio-tek Inc., (Georgia, USA). DNA standard curve was plotted from cycle threshold (Ct) values and numbers of MG derived from the culture as mentioned previously (29).

### Data Analysis

Statistical significance was determined by using SPSS software (version 24.0) through *t*-test. All the experiments were performed at least three times (*n* = 3) unless otherwise mentioned. The values are represented as mean ± standard deviations (SD) and a value of *p* < 0.05 was considered as statistically significant. The bar graphs were made by GraphPad prism (window version 6.01, San Diego, California).

## Results

### Effect of MG-infection on Antioxidant Activities in BOF Tissues

**Figure 1:**
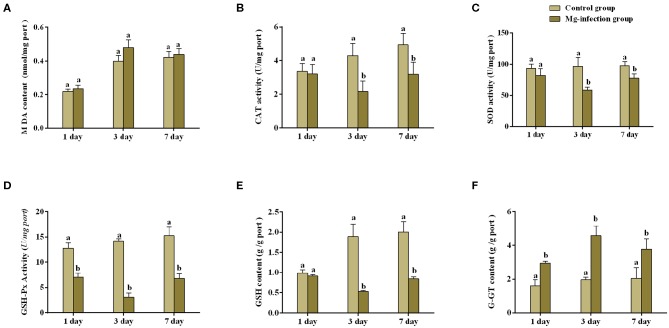
Shows the effect of MG-infection on oxidative stress-related parameters measured on 1, 3 and 7 day in chicken BOF. The assessed parameters are **(A)** MDA content **(B)** CAT activity **(C)** SOD activity **(D)** GSH-Px activity **(E)** GSH content and **(F)** G-GT content. All the bar graphs show mean results ± SD (*n* = 3). Experimental groups are represented as control group and MG-infection group. Different small letters indicate statistical significance (*p* < 0.05) between the two experimental groups at the same time point.

Figures 1A–F represents oxidative stress-related parameters in BOF tissues. It has been noted that MG-infection significantly (*p* < 0.05) reduced GSH content, SOD, CAT and GSH-Px activity at day 3 post-infection, but the reduction was not statistically significant (*p* > 0.05) at day 1 and 7 post-infection in the SOD and GSH-Px activity, except for the CAT activity and GSH content which is statistically significant at day 7 post-infection. However, G-GT content and MDA activity increased at all assessed time points. The increase in MDA activity in MG-infection group was not statistically significant compared to control group at the three time points. While, the increase in G-GT content in MG-infection group was statistically significant compared to control group at the three time points.

### Histopathological Assessment and Ultrastructural Morphology

**Figure 2:**
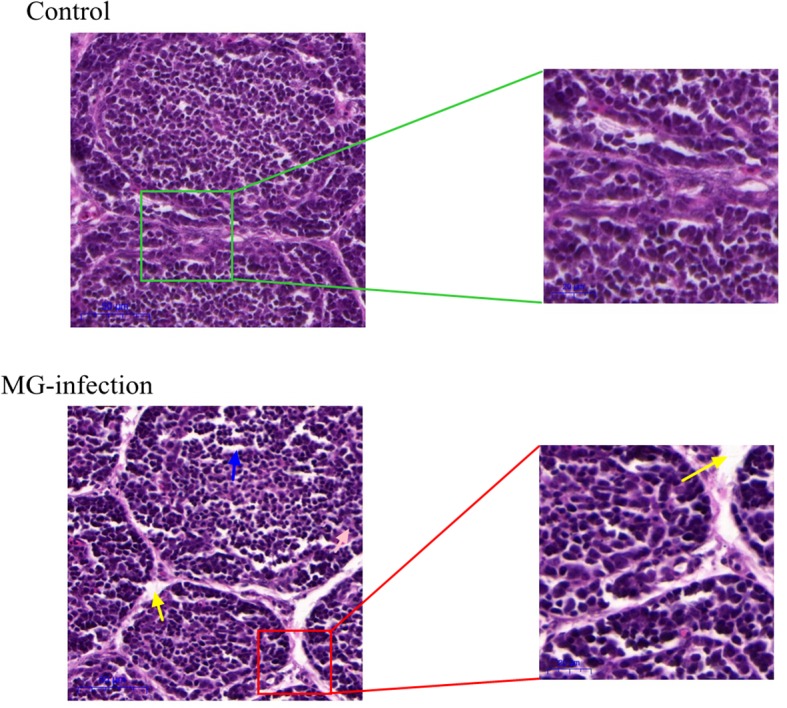
Shows the effect of MG-infection on histological examination in the BOF of chickens at day 7. BOF hematoxylin and eosin-stained sections are shown as Control group and MG-infection group (*n* = 3). The right sections in group represent 20X magnification (scale bar = 50 μm), while the zooming part is 40X magnification (scale bar = 20 μm). Blue arrow indicates vacuolation. Yellow arrow indicates increased space between bursal follicles.

**Figure 3:**
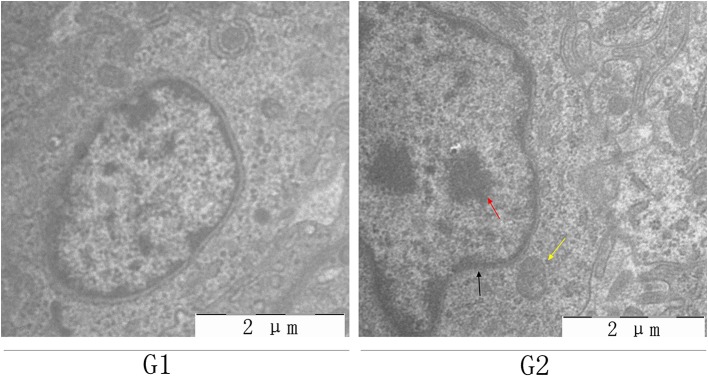
Shows the effect of MG-infection on ultrastructural changes in the BOF of chickens at day 7. BOF ultrastructural photomicrographs are shown as G1 (control group) and G2 (MG-infection group) (*n* = 3). Yellow arrow indicates mitochondrial swelling, black arrow indicates shrinkage of nuclear membrane and red arrow indicates nuclear lysis.

Histopathological and ultrastructural examinations were performed at day 7 to better understand the effects of MG infection on chicken BOF. Compared to control group, increased inflammatory cells infiltrates and necrotic lymphocytes (30, 31) were observed in the medullary region of BOF, and the disappearance of lymphocytes caused vacuolation of the medulla. In addition, no obvious pathological changes were seen in mucosal epithelial cells in MG-infection group (Figure 2). The severity of histopathological lesions in chicken BOF was shown in Table 2. Similarly, ultrastructural observation (Figure 3) revealed typical signs of apoptosis such as mitochondrial swelling, shrinkage of nuclear membrane and nuclear lysis in MG-infection group. While, most of the cells from control group showed normal morphology.

**Table 2:** Severity of histopathological lesions in chicken BOF.

**Pathological lesions**	**Control group**	**MG-infection group**
Inflammatory cell infiltration	0/15	14/15
Fragmented nuclei	1/15	12/15
Necrosis	0/15	13/15
Lymphocytes reduction	0/15	14/15

*Incidence of histopathological damage of bursa of fabricius in different groups of chickens (n = 15)*.

### The mRNA Expression and Enzyme Activities of Cytokines in Chicken BOF

**Figure 4:**
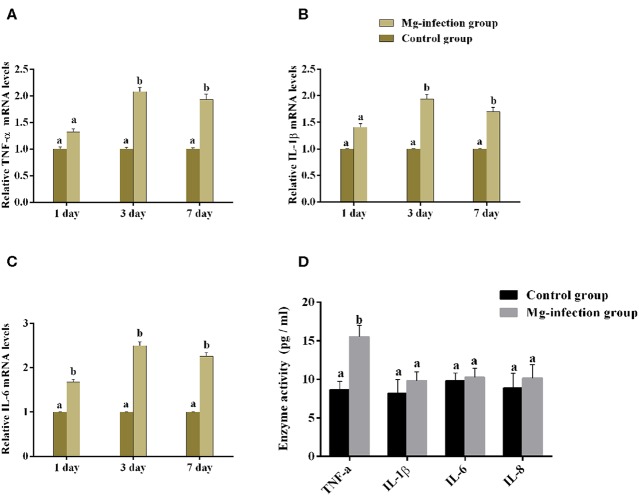
Shows the effect of MG-infection on cytokine mRNA and activities measured on 1 day, 3 day and 7 day in chicken BOF. The assessed cytokine activities are **(A)** TNF-α mRNA expression levels, **(B)** IL-1β mRNA expression level, **(C)** IL-6 mRNA expression level and **(D)** Cytokine enzyme activities. Experimental groups are represented as control group and MG-infection group. Different small letters indicate statistical significance (*p* < 0.05) between the two experimental groups at the same time point.

It is well-understood that cytokines play a crucial role in the initiation and regulation of inflammatory responses. We evaluated the mRNA expression and activities of some cytokines in BOF tissues of the two experimental groups [shown in Figures 4A–D]. Compared to control group, the mRNA expression level of TNF-α, IL-1β and IL-6 (Figures 4A–C) were increased in MG-infection group at the three time points. There was a statistically significant difference noted in TNF-α, IL-1β and IL-6 mRNA expression at day 3 and day 7 compared to day 1 post-infection. Cytokine enzyme activities were detected at day 7 (Figure 4D). Notably, TNF-α enzyme activity was significantly (*p* < 0.05) increased in BOF tissues in MG-infection group. While, the increase in IL-1β, IL-6, and IL-8 activities were not statistically significant (*p* > 0.05) in MG-infection group compared to control group.

### Effect of MG-infection on NO Content and iNOS mRNA, Enzyme Activity and Protein Expression Level in Chicken BOF

**Figure 5:**
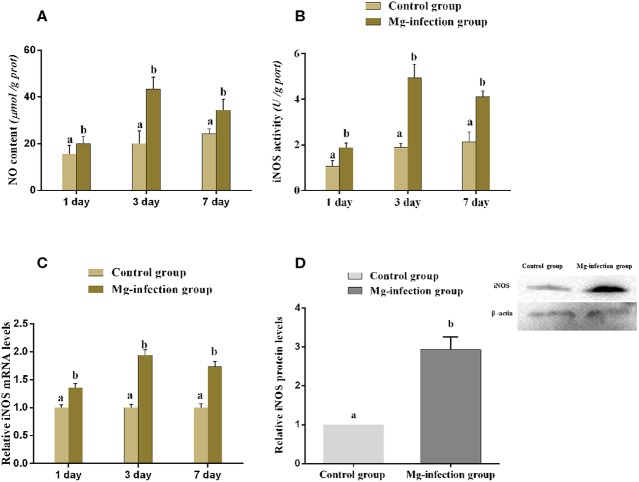
Shows the effect of MG-infection on **(A)** NO content, **(B)** iNOS activity **(C)** iNOS mRNA expression level and **(D)** iNOS protein expression level in chicken. Experimental groups are represented as control group and MG-infection group. Different small letters indicate statistical significance (*p* < 0.05) between the two experimental groups at the same time point.

Figure 5 displays the effect of MG-infection on NO content and iNOS mRNA, enzyme activity and protein expression level in BOF tissues. NO content (Figure 5A) was increased in BOF tissues at day 1, day 3, and day 7 post-infection in MG-infection group compared to control group. Compared to control group, iNOS activity and mRNA expression (Figures 5B,C) were significantly increased at the three time points in MG-infection group. In addition, iNOS protein expression (Figure 5D) showed the same trend as mRNA and enzyme activity at day 7. There was a significant increase in iNOS protein expression level in MG-infection group compared to control group. The original blots have been shown in Supplementary Material (Data Sheet 1).

### Expression of Apoptotic Genes and TUNEL Assay in Chicken BOF

**Figure 6:**
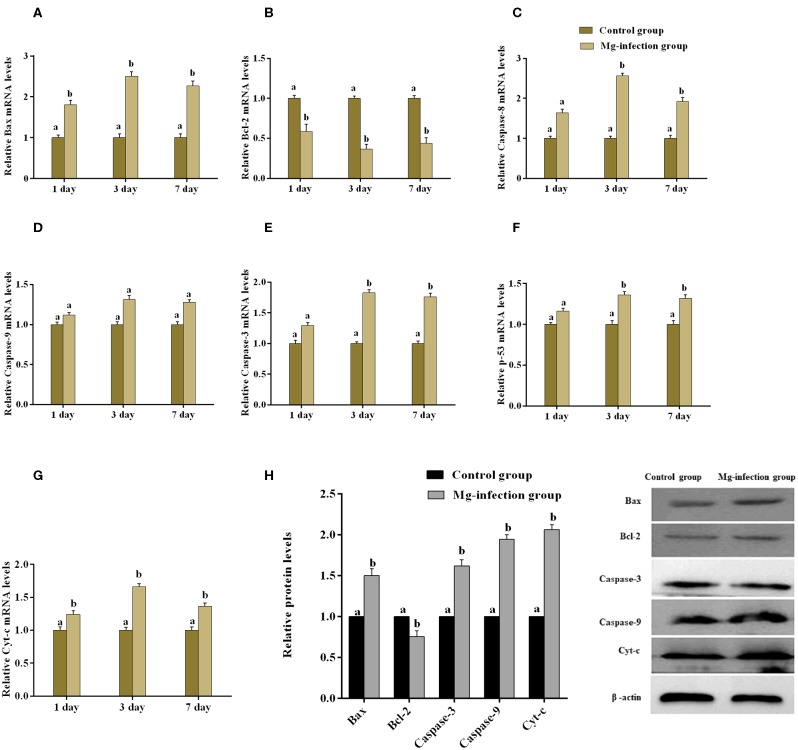
shows the effect of MG-infection on apoptosis-related genes mRNA **(A–G)** and protein expression **(H)** in chicken BOF. Experimental groups are represented as control group and MG-infection group. Different small letters indicate statistical significance (*p* < 0.05) between the two experimental groups at the same time point.

**Figure 7:**
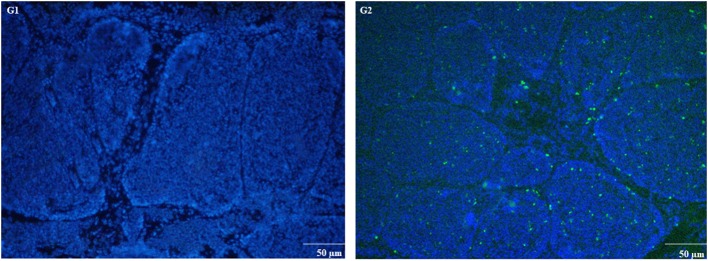
TUNEL assay was used to measure apoptosis in chicken BOF tissues at day 7. Experimental groups are represented as (G1) control group and (G2) MG-infection group (*n* = 3). Large numbers of positive stained apoptotic nuclei were seen in MG-infection group (scale bar = 50 μm).

The expression of apoptosis-related genes both at mRNA and protein level is shown in Figures 6A–H. Compared to control group, MG-infection significantly enhanced the mRNA expression of apoptosis-related genes (Figures 6A–G). However, the mRNA expression of anti-apoptotic Bcl2 gene was significantly (*p* < 0.05) reduced at 3- and 7-day post-infection. In addition, protein expression results showed similar trends as mRNA expression (Figure 6H). There was a significant increase in protein expression of apoptosis-related genes, with the exception of Bcl2, which showed significant downregulation in BOF tissues in MG-infection group compared to control group. TUNEL assay (Figure 7) further confirmed that MG-infection induced apoptosis in chicken BOF. Compared to control group, increased number of positive stained nuclei were observed in chicken BOF tissues in MG-infection group. These results suggested that MG-infection remarkably increased the number of apoptotic cells in chicken BOF and could impair its immune function.

### Effect of MG on CD8^+^ Lymphocytes

**Figure 8:**
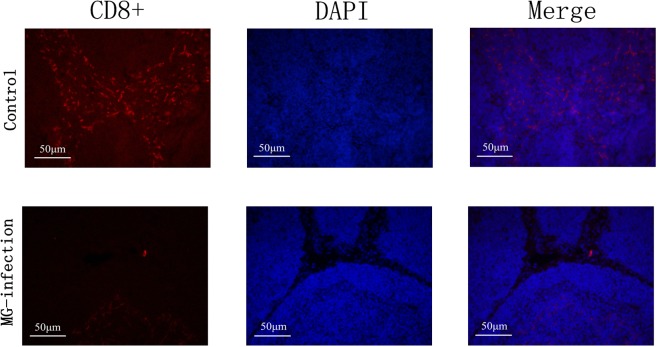
Immunofluorescence microscopy showed that MG-infection significantly reduced the number of CD8^+^ cells in chicken BOF at day 7. The photomicrographs are represented as CD8^+^, DAPI and Merge in each group. Whereas, the groups are represented as control group and MG-infection group. The photomicrographs were taken at 400X magnification (*n* = 3).

The results of immunofluorescence microscopic examination were shown in Figure 8. It has been examined that MG infection significantly reduced the number of CD8^+^ cells in MG-infection group compared to control group at day 7 post-infection. The depletion of CD8^+^ cells in chicken BOF could be due to the induction of apoptosis by MG.

### Quantification of Bacterial Load in Chicken BOF

**Figure 9:**
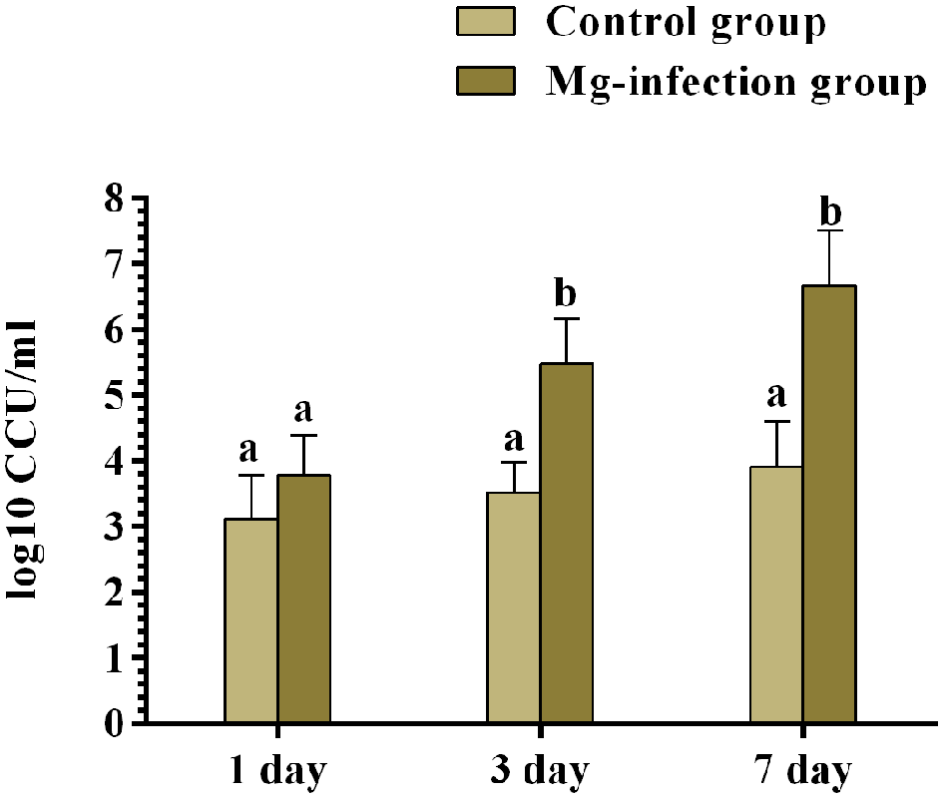
The copy numbers of MG challenge strain were determined by qRT-PCR at day 1, 3 and 7 post-infection. Bars represent the mean value of three replicates and error bars show the standard deviation (SD). Different small letters indicate statistical significance (*p* < 0.05) between the two experimental groups at the same time point.

The results of bacterial quantification were shown in Figure 9. The copy numbers of MG were significantly higher in MG-infection group compared to control group at 3- and 7-day post-infection. While, the increase in copy numbers at day 1 was not statistically significant compared to control group.

## Discussion

The research on MG-infection has been carried out for several years, but the understanding of immune impairment remains elusive because of the complex inflammatory responses during MG infection. Several researchers investigated the effect of MG infection on lymphoid organs such as thymus, spleen and BOF (32, 33). In the present study, the effects of MG-infection on structural integrity and immune function of chicken BOF were scrutinized. Oxidative stress is increased during many pathogenic infections (34). Similarly, our data also showed that MG-infection induced oxidative stress and significantly reduced antioxidant activities at day 3 and day 7 post-infection. In addition, MG-infection impaired the structural integrity of chicken BOF. The histopathological and ultrastructural examination showed clear abnormal morphological signs in MG-infection group. Based on these findings, it can be speculated that MG-infection could disturb the normal function of chicken lymphoid organs. Similarly, another study reported lymphocytolysis and depletion in the medullary region in BOF during MG-infection (33). While, the exact cause of MG-induced immune impairment and its underlying mechanism in chicken is still not completely understood.

Studies reported that a continuous release of inflammatory cytokines plays a critical role in effective immune responses against pathogenic infections. It has been demonstrated that MG lipid associated membrane proteins (LAMPs) mediated inflammatory responses in chicken tracheal epithelial cells through nuclear factor kappa B (NF-κB) signaling pathway resulting in the downstream stimulation of several pro-inflammatory cytokines and chemokines (35). Moreover, cytokine microenvironment distinguishes the polarization of T cells, macrophages, and associated with the production of new antibodies (36). Notably, it has been reported that the activation and recruitment of inflammatory cells are involved in the crux of MG pathogenesis (37). Due to the complex mechanisms of cytokines involved in the proliferation and activation of macrophages and other immune cells (38–41), the underlying mechanisms in the activation and proliferation of these cells are still not reported. In the present study, we found that the expression level of pro-inflammatory cytokines increased at all assessed time points in BOF tissues following MG-infection. A significant (*p* < 0.05) increase has been noted in TNF-α level at day 3 and 7 post-infection. Hence, it is speculated that apoptosis induced by MG-infection could be due the increased TNF-α level in BOF tissues. Similarly, another study also reported that increased TNF-α level induced apoptosis in lymphoid organs (42). However, further molecular studies are needed to understand the detailed mechanism of MG infection-induced apoptosis and increased level of TNF-α in chicken BOF.

Systemic inflammation such as sepsis, bacterial lipid polysaccharide (LPS) administration or pathogen invasion induced lymphoid organ apoptosis (43). Researchers reported that the amount of apoptosis is proportional to the severity of disease pathogeneses and excessive apoptosis may lead to malfunctioning, atrophy and/or complete organ failure (44, 45). In the present study, changes in genes expression levels such as Bax, Caspases-3, Caspases-9, Bcl2, p53, and Cytochrome-C suggested that MG-infection induced apoptosis in chicken BOF. TUNEL assay results revealed positive stained nuclei in BOF of chickens following MG-infection. Moreover, ultrastructural microscopic analysis further confirmed the phenomenon of MG-induced apoptosis. Swollen and deformed mitochondria were found in the BOF of chickens infected with MG. Moreover, there are several factors such as the infection dose, time, health status of the chickens, species and route of infection that are involved in the severity, and induction of apoptosis during MG-infection. Another possible evidence may be the increased oxidative stress or TNF-α alone or in combination which induced apoptosis in chicken BOF. Previous studied demonstrated that both oxidative stress and inflammation are the crucial factors that induced apoptosis through different cellular pathways. Growing evidences showed that oxidative stress induced apoptosis through both mitochondrial-dependent or independent cell signaling pathways (46–48). However, it is worthy to mention that the molecular mechanism among inflammation, oxidative stress and the induction of apoptosis in chicken BOF is still elusive. Although researchers reported that there are phase variable surface lipoproteins in MG that helps to escape it from the host immune system (49).

Studies demonstrated that T-lymphocytes passed through a series of recruitment stages and culling process such as apoptosis and undergo differentiation into CD4^+^ or CD8^+^ single positive cells (50). While, the pathogenic infections may adversely affect the recruitment process (51, 52). Manafi et al., reported that MG infection damage the immune function of chicken BOF (33). In the present study, immunofluorescence microscopy results showed that MG-infection caused depletion of CD8^+^ cells in chicken BOF. We speculate that this depletion could be due to extensive apoptosis induced by MG. These findings are crucial to identify the molecular mechanisms involved in host immune system destruction rather than beneficial to host immune responses and may be important in the targeted immune therapy and vaccines development. However, further studies are needed to scrutinize the complex relationship among induction of apoptosis, inflammation and immune impairment in chicken BOF.

## Conclusion

**Figure 10:**
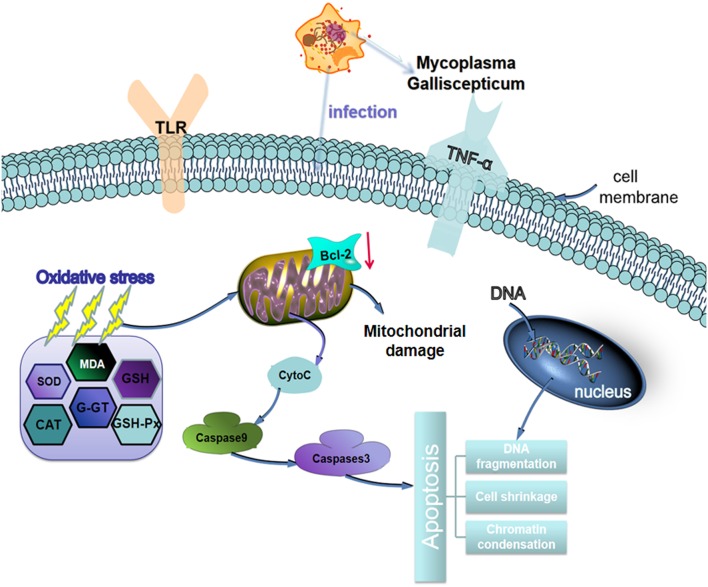
Schematic representation of MG-infection-induced oxidative stress and apoptosis in chicken BOF tissues. The arrows show connection or association between signaling molecules.

In conclusion, MG-infection induced oxidative stress and caused alteration in cytokines activities. The structural integrity of BOF was impaired and abnormal morphological signs were observed in MG-infection group. In addition, increased number of apoptotic cells were observed in BOF of chickens infected with MG. The schematic diagram of MG induced oxidative and apoptosis is shown in Figure 10. Taken together, the study gives an insight into immune impairment in chickens and lay a foundation for further studies to investigate the detail molecular mechanism behind impair immune functions.

## Data Availability Statement

All datasets generated for this study are included in the article/Supplementary Material.

## Ethics Statement

The animal study was reviewed and approved by Institutional Animal Care and Use Committee of Northeast Agricultural University.

## Author Contributions

JL and JW design the paper. WZ and YL performed experiments. QZ, SW, and ZW provided help during experiments. MI made critical revisions to the paper and contributed to paper writing.

## Conflict of Interest

The authors declare that the research was conducted in the absence of any commercial or financial relationships that could be construed as a potential conflict of interest.

## Supplementary Material

Data Sheet 1 The blots represent the expression of different proteins in chicken BOF.

## References

[1] Lu Z Xie D Chen Y Tian E Muhammad I Chen X. TLR2 mediates autophagy through ERK signaling pathway in *Mycoplasma* gallisepticum-infected RAW264.7 cells. Mol Immunol. (2017) 87:161–70. 10.1016/j.molimm.2017.04.013 28478286

[2] Nunoya T Tajima M Yagahashi T Sannai S. Natural case of salpingitis apparently caused by *Mycoplasma gallisepticum* in chickens. Avian Pathol. (1997) 26:391–8. 10.1080/03079459708419221 18483915

[3] Gaunson JE Philip CJ Whithear KG Browning GF. The cellular immune response in the tracheal mucosa to *Mycoplasma Gallisepticum* in vaccinated and unvaccinated chickens in the acute and chronic stages of disease. Vaccine. (2006) 24:2627–33. 10.1016/j.vaccine.2005.12.008 16406173

[4] Beaudet J Tulman ER Pflaum K Liao X Kutish GF Szczepanek SM. Transcriptional profiling of the chicken tracheal response to virulent *Mycoplasma Gallisepticum* strain rlow. Infect Immun. (2017) 85:e00343–17. 10.1128/IAI.00343-17 28739827 PMC5607433

[5] Jacob R Branton SL Evans JD Leigh SA Peebles ED. Effects of live and killed vaccines against *Mycoplasma Gallisepticum* on the performance characteristics of commercial layer chickens. Poult Sci. (2014) 93:1403–9. 10.3382/ps.2013-03748 24879690

[6] Mohammed J Frasca S Jr Cecchini K Rood D Nyaoke AC Geary SJ Chemokine and cytokine gene expression profiles in chickens inoculated with *Mycoplasma Gallisepticum* strains Rlow or GT5. Vaccine. (2007) 25:8611–21. 10.1016/j.vaccine.2007.09.057 18006123

[7] Tryon W Baseman JB Pathogenic determinants and mechanisms. In: Maniloff J McElhaney RN Finch LR Baseman JB (editors). Mycoplasmas: Molecular Biology and Pathogenesis. American Society for Microbiology, Washington, DC: ASM Press (1992). p. 457–71

[8] Xu Y Li H Chen W Yao X Xing Y Wang X. *Mycoplasma Hyorhinis* activates the NLRP3 inflammasome and promotes migration and invasion of gastric cancer cells. PLoS ONE. (2013) 8:e77955. 10.1371/journal.pone.0077955 24223129 PMC3819327

[9] Li H Horke S Förstermann U. Oxidative stress in vascular disease and its pharmacological prevention. Trends Pharmacol Sci. (2013) 34:313–9. 10.1016/j.tips.2013.03.007 23608227

[10] Rochette L Lorin J Zeller M Guilland JC Lorgis L Cottin Y. Nitric oxide synthase inhibition and oxidative stress in cardiovascular diseases: possible therapeutic targets?. Pharmacol. Ther. (2013) 140:239–57. 10.1016/j.pharmthera.2013.07.004 23859953

[11] Goldszmid RS Trinchieri G. The price of immunity. Nat Immunol. (2012) 13:932–8. 10.1038/ni.2422 22990891

[12] Gostner JM Becker K Ueberall F Fuchs D. The good and bad of antioxidant foods: an immunological perspective. Food Chem Toxicol. (2015) 80:72–9. 10.1016/j.fct.2015.02.012 25698357

[13] Li WJ Nie SP Peng XP Liu XZ Li C Chen Y. *Ganoderma Atrum* polysaccharide improves age-related oxidative stress and immune impairment in mice. J Agric Food Chem. (2012) 60:1413–8. 10.1021/jf204748a 22264032

[14] Li WJ Li L Zhen WY Wang LF Pan M Lv JQ. Ganoderma atrum polysaccharide ameliorates ROS generation and apoptosis in spleen and thymus of immunosuppressed mice. Food Chem Toxicol. (2017) 99:199–208. 10.1016/j.fct.2016.11.033 27913287

[15] Srikumar R Parthasarathy NJ Manikandan S Narayanan GS Sheeladevi R. Effect of triphala on oxidative stress and on cell-mediated immune response against noise stress in rats. Mol Cell Biochem. (2006) 283:67–74. 10.1007/s11010-006-2271-0 16444587

[16] Zhang Y Zhou Y Sun G Li K Li Z Su A. Transcriptome profile in bursa of fabricius reveals potential mode for stress-influenced immune function in chicken stress model. BMC Genomics. (2018) 19:918. 10.1186/s12864-018-5333-2 30545299 PMC6293626

[17] Paramithiotis E Michael JHR. B cell emigration directly from the cortex of lymphoid follicles in the bursa of fabricius. Eur J Immunol. (1994) 24:458–63. 10.1002/eji.1830240229 8299695

[18] Li R Kou X Tian J Meng Z Cai Z Cheng F. Effect of sulfur dioxide on inflammatory and immune regulation in asthmatic rats. Chemosphere. (2014) 112:296–304. 10.1016/j.chemosphere.2014.04.065 25048919

[19] Hu X Chi Q Wang D Chi X Teng X Li S. Hydrogen sulfide inhalation-induced immune damage is involved in oxidative stress, inflammation, apoptosis and the Th1/Th2 imbalance in broiler bursa of fabricius. Ecotoxicol Environ Saf . (2018) 164:201–9. 10.1016/j.ecoenv.2018.08.029 30118953

[20] Ishfaq M Chen C Bao J Zhang W Wu Z Wang J. Baicalin ameliorates oxidative stress and apoptosis by restoring mitochondrial dynamics in the spleen of chickens via the opposite modulation of NF-κB and Nrf2/HO-1 signaling pathway during *Mycoplasma Gallisepticum* infection. Poult Sci. (2019) 98:6296–310. 10.3382/ps/pez406 31376349 PMC8913776

[21] Wu Z Ding L Bao J Liu Y Zhang Q Wang J. Co-infection of *Mycoplasma Gallisepticum* and *Escherichia Coli* triggers inflammatory injury involving the IL-17 signaling pathway. Front Microbiol. (2019) 10:2615. 10.3389/fmicb.2019.02615 31803158 PMC6872679

[22] Wang J Yi M Chen X Muhammad I Liu F Li R. Effects of colistin on amino acid neurotransmitters and blood-brain barrier in the mouse brain. Neurotoxicol Teratol. (2016) 55:32–7. 10.1016/j.ntt.2016.03.004 27018023

[23] Livak KJ Schmittgen TD. Analysis of relative gene expression data using real time quantitative PCR and the 2 [-Delta Delta C (T)] method. Methods. (2001) 25:402–8. 10.1006/meth.2001.1262 11846609

[24] Lu Z Miao Y Muhammad I Tian E Hu W Wang J. Colistin-induced autophagy and apoptosis involves the JNK-Bcl2-Bax signaling pathway and JNK-p53-ROS positive feedback loop in PC-12 cells. Chem Biol Interact. (2017) 277:62–73. 10.1016/j.cbi.2017.08.011 28842171

[25] Jiao W Han Q Xu Y Jiang H Xing H Teng X. Impaired immune function and structural integrity in the gills of common carp (*Cyprinus Carpio L*.) caused by chlorpyrifos exposure: through oxidative stress and apoptosis. Fish Shellfish Immunol. (2019) 86:239–45. 10.1016/j.fsi.2018.08.060 30176333

[26] Hu X Chi Q Liu Q Wang D Zhang Y Li S. Atmospheric H2S triggers immune damage by activating the TLR-7/MyD88/NF-κB pathway and NLRP3 inflammasome in broiler thymus. Chemosphere. (2019) 237:124427. 10.1016/j.chemosphere.2019.124427 31352103

[27] Bao J Wu Z Ishfaq M Miao Y Li R Clifton AC. Comparison of experimental infection of normal and immunosuppressed chickens with *Mycoplasma Gallisepticum*. J Comp Pathol. (2020) 175:5–12. 10.1016/j.jcpa.2019.12.001 32138843

[28] Mekkes DR Feberwee A. Real-time polymerase chain reaction for the qualitative and quantitative detection of *Mycoplasma Gallisepticum*. Avian Pathology. (2005) 34:348–54. 10.1080/03079450500179954 16147572

[29] Grodio JL Dhondt KV O'Connell PH Schat KA Detection and quantification of *Mycoplasma Gallisepticum* genome load in conjunctival samples of experimentally infected house finches (*Carpodacus Mexicanus*) using realtime polymerase chain reaction. Avian Pathology. (2008) 37:385–91. 10.1080/03079450802216629 18622854

[30] Carvallo FR French RA Gilbert-Marcheterre K Risatti G Dunn JR Forster F. Mortality of one-week-old chickens during naturally occurring Marek's disease virus infection. Vet Pathol. (2011) 48:993–8. 10.1177/0300985810395727 21239693

[31] Nakamura K Imada Y Maeda M. Lymphocytic depletion of bursa of fabricius and thymus in chickens inoculated with *Escherichia Coli*. Vet Pathol. (1986) 23:712–7. 10.1177/030098588602300610 3544459

[32] Lain KM Lin W Resistance of chickens immunized against *Mycoplasma Gallisepticum* is mediated by bursal dependent lymphoid cells. Vet Microbiol. (1984) 9:509–14. 10.1016/0378-1135(84)90072-5 6495612

[33] Manafi M Pirany N Noor Ali M Hedayati M Khalaji S Yari M. Experimental pathology of T-2 toxicosis and *Mycoplasma* infection on performance and hepatic functions of broiler chickens. Poult Sci. (2015) 94:1483–92. 10.3382/ps/pev115 25910901

[34] Poggi C Dani C. Sepsis and oxidative stress in the newborn: from pathogenesis to novel therapeutic targets. Oxid Med Cell Longev Oxid Med Cell Longev. (2018) 2018:9390140. 10.1155/2018/9390140 30174784 PMC6098933

[35] Majumder S Zappulla F Silbart LK *Mycoplasma Gallisepticum* lipid associated membrane proteins up-regulate inflammatory genes in chicken. Tracheal epithelial cells via TLR-2 ligation through an NF-kB dependent pathway. PLoS ONE. 9:e112796 10.1371/journal.pone.0112796 PMC4234737 25401327

[36] Bielinska AU O'Konek JJ Janczak KW Baker JR Jr. Immunomodulation of TH2 biased immunity with mucosal administration of nanoemulsion adjuvant. Vaccine. (2016) 34:4017–24. 10.1016/j.vaccine.2016.06.043 27317451 PMC4962973

[37] Gaunson JE Philip CJ Whithear KG Browning GF. Lymphocytic infiltration in the chicken trachea in response to *Mycoplasma Gallisepticum* infection. Microbiology. (2000) 146:1223–9. 10.1099/00221287-146-5-1223 10832650

[38] Majumder S Silbart LK Interaction of *Mycoplasma Gallisepticum* with chicken tracheal epithelial cells contributes to macrophage chemotaxis and activation. Infect Immun. (2015) 2:266–74. 10.1128/IAI.01113-15 PMC4693983 26527215

[39] Razin S Yogev D Naot Y. Molecular biology and pathogenicity of *Mycoplasma*s. Microbiol Mol Biol Rev. (1998) 62:1094–156. 10.1128/MMBR.62.4.1094-1156.1998 9841667 PMC98941

[40] Rottem S. Interaction of Mycoplasmas with host cells. Physiol Rev. (2003) 83:417–32. 10.1152/physrev.00030.2002 12663864

[41] Chambaud I Wroblewski H Blanchard A. Interactions between *Mycoplasma lipoproteins* and the host immune system. Trends Microbiol. (1999) 7:493–9. 10.1016/S0966-842X(99)01641-8 10603485

[42] Zang GQ Zhou XQ Yu H Xie Q Zhao GM Wang B. Effect of hepatocyte apoptosis induced by TNF-α on acute severe hepatitis in mouse models. World J Gastroenterol. (2000) 6:688–92. 10.3748/wjg.v6.i5.688 11819675 PMC4688844

[43] Kato Y Morikawa A Sugiyama T Koide N Jiang GZ Takahashi K. Role of tumor necrosis factor-alpha and glucocorticoid on lipopolysaccharide (LPS)-induced apoptosis of thymocytes. FEMS Immunol Med Microbiol. (1995) 12:195–204. 10.1111/j.1574-695X.1995.tb00192.x 8745003

[44] Kato Y Morikawa A Sugiyama T Koide N Jiang GZ Lwin T. Augmentation of lipopolysaccharide-induced thymocyte apoptosis by interferon-gamma. Cell Immunol. (1997) 177:103–8. 10.1006/cimm.1997.1103 9178636

[45] Harrison L Brown C Afonso C Zhang J Susta L. Early occurrence of apoptosis in lymphoid tissues from chickens infected with strains of newcastle disease virus of varying virulence. J Comp Pathol. (2011) 145:327–35. 10.1016/j.jcpa.2011.03.005 21511269

[46] Jin X Xu Z Zhao X Chen M Xu S. The antagonistic effect of selenium on lead-induced apoptosis via mitochondrial dynamics pathway in the chicken kidney. Chemosphere. (2017) 180:259–66. 10.1016/j.chemosphere.2017.03.130 28411542

[47] Sinha K Das J Pal PB Sil PC. Oxidative stress: the mitochondria-dependent and mitochondria-independent pathways of apoptosis. Arch Toxicol. (2013) 87:1157–80. 10.1007/s00204-013-1034-4 23543009

[48] Wang L Cao J Chen D Liu X Lu H Liu Z. Role of oxidative stress, apoptosis, and intracellular homeostasis in primary cultures of rat proximal tubular cells exposed to cadmium. Biol Trace Elem Res. (2009) 127:53. 10.1007/s12011-008-8223-7 18802671

[49] Matyushkina D Pobeguts O Butenko I Vanyushkina A Anikanov N Bukato O. Phase Transition of the bacterium upon invasion of a host cell as a mechanism of adaptation: a *Mycoplasma Gallisepticum* model. Sci Rep. (2016) 6:35959. 10.1038/srep35959 27775027 PMC5075909

[50] Fowlkes BJ Edison L Mathieson BJ Chused TM. Early Tlymphocytes. Differentiation *in vivo* of adult intrathymic precursor cells. J Exp Med. (1985) 162:802–22. 10.1084/jem.162.3.802 2863322 PMC2187800

[51] Anderson G Jenkinson EJ. Lymphostromal interactions in thymic development and function. Nat Rev Immunol. (2001) 1:31–40. 10.1038/35095500 11905812

[52] Coutinho A Caramalho I Seixas E Demengeot J Thymic commitment of regulatory T cells is a pathway of TCR-dependent selection that isolates repertoires undergoing positive or negative selection. Curr Topics Microbiol Immunol. (2005) 293:43–71. 10.1007/3-540-27702-1_3 15981475

