# Supplementary figures and images for: Mycoplasma gallisepticum Infection Impaired the Structural Integrity and Immune Function of Bursa of Fabricius in Chicken: Implication of Oxidative Stress and Apoptosis
Source: Front Vet Sci. 2020 Apr 24;7:225. doi: 10.3389/fvets.2020.00225 (PMC7193947; doi:10.3389/fvets.2020.00225)

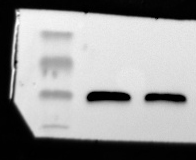

Supplement: Data Sheet 1 — The blots represent the expression of different proteins in chicken BOF. [file Data_Sheet_1.ZIP › Original blots for apoptosis/B-actin.jpg]

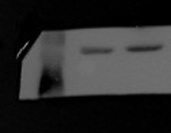

Supplement: Data Sheet 1 — The blots represent the expression of different proteins in chicken BOF. [file Data_Sheet_1.ZIP › Original blots for apoptosis/Bax.jpg]

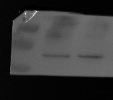

Supplement: Data Sheet 1 — The blots represent the expression of different proteins in chicken BOF. [file Data_Sheet_1.ZIP › Original blots for apoptosis/Bcl2.jpg]

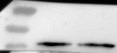

Supplement: Data Sheet 1 — The blots represent the expression of different proteins in chicken BOF. [file Data_Sheet_1.ZIP › Original blots for apoptosis/Casp-3.jpg]

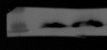

Supplement: Data Sheet 1 — The blots represent the expression of different proteins in chicken BOF. [file Data_Sheet_1.ZIP › Original blots for apoptosis/Casp-9.jpg]

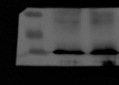

Supplement: Data Sheet 1 — The blots represent the expression of different proteins in chicken BOF. [file Data_Sheet_1.ZIP › Original blots for apoptosis/Cyt-C.jpg]

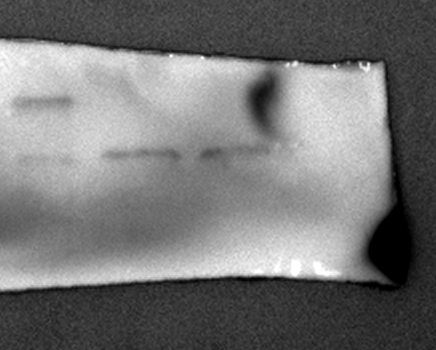

Supplement: Data Sheet 1 — The blots represent the expression of different proteins in chicken BOF. [file Data_Sheet_1.ZIP › Original blots for iNOS and b-actin/b-actin.tif]

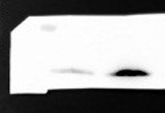

Supplement: Data Sheet 1 — The blots represent the expression of different proteins in chicken BOF. [file Data_Sheet_1.ZIP › Original blots for iNOS and b-actin/iNOS.jpg]
